# Identification of a novel form of caspase-independent cell death triggered by BH3-mimetics in diffuse large B-cell lymphoma cell lines

**DOI:** 10.1038/s41419-024-06652-3

**Published:** 2024-04-15

**Authors:** Nahide Yildirim, Lakshmi Sarojam, Victoria M. Smith, Nadja M. Pieper, Marius Anders, Ross A. Jackson, Dominik C. Fuhrmann, Vinzenz Särchen, Daniela Brücher, Andreas Weigert, Martin J. S. Dyer, Meike Vogler

**Affiliations:** 1 Institute for Experimental Pediatric Hematology and Oncology, Goethe University Frankfurt, Frankfurt, Germany; 2 The Ernest and Helen Scott Haematological Research Institute, Leicester Cancer Research Centre, University of Leicester, Leicester, UK; 3 Faculty of Medicine, Institute of Biochemistry I, Goethe University Frankfurt, Frankfurt am Main, Germany; 4 German Cancer Consortium (DKTK) partner site Frankfurt/Mainz, a partnership between DKFZ and University Hospital Frankfurt, Frankfurt, Germany; 5 University Cancer Center Frankfurt (UCT), University Hospital Frankfurt, Goethe-University Frankfurt, Frankfurt, Germany

**Keywords:** Targeted therapies, Transcriptional regulatory elements

## Abstract

BH3-mimetics represent promising anti-cancer agents in tumors that rely on the anti-apoptotic function of B-Cell Lymphoma 2 (BCL2) proteins, particularly in leukemia and lymphoma cells primed for apoptosis. Mechanistically, BH3-mimetics may displace pro-apoptotic binding partners thus inducing BAX/BAK-mediated mitochondrial permeabilization followed by cytochrome *c* release, activation of the caspase cascade and apoptosis. Here, we describe a novel mode of caspase-independent cell death (CICD) induced by BH3-mimetics in a subset of diffuse large B-cell lymphoma (DLBCL) cells. Of note, rather than occurring via necroptosis, CICD induced immediately after mitochondrial permeabilization was associated with transcriptional reprogramming mediated by activation of c-Jun N-terminal Kinase (JNK) signaling and Activator Protein 1 (AP1). Thereby, CICD resulted in the JNK/AP1-mediated upregulation of inflammatory chemokines and increased migration of cytotoxic Natural Killer (NK) cells. Taken together, our study describes a novel mode of CICD triggered by BH3-mimetics that may alter the immune response towards dying cells.

## Introduction

Many tumor types are characterized by high expression of anti-apoptotic BCL2 family proteins. BCL2 itself is often overexpressed in lymphoid malignancies, either due to copy number gains or the t(14;18)(q21.3;q32.3) chromosomal translocation that drives expression of BCL2 under the immunoglobulin heavy-chain enhancer [1]. The anti-apoptotic BCL2 proteins are held in check by the pro-apoptotic family members, in particular the BCL2 homology domain 3 (BH3)-only proteins, which are upregulated upon cellular stress and neutralize the anti-apoptotic BCL2 proteins. BH3-only proteins compete with the multidomain proteins BAX and BAK for the binding of the anti-apoptotic BCL2 proteins. Once activated, BAX and BAK form pores in the outer mitochondrial membrane, through which cytochrome *c* is released. Thus, BAX and BAK are kept in an inactive state through sequestration by the anti-apoptotic BCL2 proteins. Of note, recent reports have highlighted the importance of the BAX/BAK macropores also for the release of mitochondrial DNA (mtDNA) [2] and identified an essential link between the apoptotic regulatory network and the activation of the cGAS/STING pathway during cellular senescence [3].

The mechanistic understanding of the interactions within the BCL2 protein family and its key role in regulating mitochondrial outer membrane permeabilization (MOMP) facilitated the discovery of a hydrophobic groove on the surface of the anti-apoptotic BCL2 proteins that is targetable by small-molecule inhibitors, leading to the development of BH3-mimetics that bind with high affinity and displace pro-apoptotic binding partners [4–6]. BH3-mimetics have been shown to induce apoptosis in a variety of cancer cells and under different cellular conditions [7, 8]. The selective inhibitor of BCL2, ABT199 (venetoclax), has been approved for the treatment of chronic lymphocytic leukemia (CLL) and acute myeloid leukemia (AML) [9, 10], highlighting the potential of this targeted treatment therapy.

Besides apoptosis, several other forms of cell death have been described that follow tightly structured and regulated signaling events [11, 12]. In contrast to apoptosis, which is dependent on active caspases, these non-apoptotic forms of regulated cell death can occur in the absence of active caspases and can therefore be classified as caspase-independent cell death (CICD). Although the relevance of CICD in vivo is still under investigation, the classification of the main initiator caspases-8 and -9 by the Cancer Gene Census as Consensus Tier 1 and 2 genes, respectively, highlights that cancer cells employ diverse evasion strategies to resist apoptotic cell death [13]. One of the best characterized non-apoptotic cell death pathways is necroptosis, a programmed form of necrosis involving the activation of Receptor-interacting protein kinase 1 (RIPK1), the oligomerization of the mixed lineage kinase domain-like protein (MLKL) in the plasma membrane and subsequent rupture of the plasma membrane [14]. Of note, necroptosis may also be induced by MOMP following BH3-mimetic treatment [15]. In that scenario, MOMP in the absence of caspase activation leads to the degradation of Inhibitor of Apoptosis Proteins (IAPs), accumulation of NF-κB-inducing kinase (NIK), activation of NF-κB signaling, increased Tumor necrosis factor alpha (TNFα) synthesis and necroptosis.

Another form of CICD is ferroptosis triggered by the accumulation of lipid peroxides on cellular membranes and regulated by GPX4. Although the role of mitochondria in ferroptosis is under debate, mitochondria are affected during ferroptosis and typically appear shrunken with increased matrix density [16]. Mitochondria are also the main source of Reactive Oxygen Species (ROS). They are highly dynamic organelles with a constant turnover through mitophagy, the selective degradation of mitochondria in autophagosomes, which may also lead to cell death [17, 18]. Anti-apoptotic BCL2 proteins may suppress mitophagy by inhibiting Parkin translocation to depolarized mitochondria [19]. In this context, BH3-mimetics targeting the anti-apoptotic BCL2 proteins may promote mitophagy [20–22].

Here, we observed a rapid form of CICD that occurred in some DLBCL cells exposed to BH3-mimetics. Induction of CICD leads to the activation of JNK/AP1 signaling pathways resulting in massive transcriptional reprogramming and upregulation of proinflammatory cytokines. The potential physiologic importance of this novel form of cell death is demonstrated by an increased migration of cytotoxic NK cells under CICD conditions, indicating that induction of CICD rather than apoptosis may activate an anti-tumor immune response.

## Methods

### Chemicals

Cells were treated with BH3-mimetics (Selleck Chemicals, Houston, TX), the caspase inhibitors zVAD.fmk and QVD.OPh (Bachem, Switzerland). To analyze secretion of chemokines, chemokine bead array (#740930, BioLegend, San Diego, CA) was used according to manufacturer’s instructions.

### Analysis of viability, cell death or mitochondrial membrane potential (MMP)

Analysis of cell viability after drug treatment was done with CellTiter-Glo® (CTG) assay (Promega, Madison, WI). Alternatively, dead cells were analyzed by flow cytometry after staining with AnnexinV-FITC and propidium iodide (PI). Loss of MMP was analyzed by staining with tetramethyl-rhodamine methylester (TMRM) and flow cytometry at FACS Canto II as described previously [23].

### Electron microscopy (EM) and ImageStream analysis

SU-DHL-6 cells were exposed to BH3-mimetics (3 μM ABT199 or 100 nM S63845) for 8 h in the presence of 10 μM QVD.OPh before fixation and resin embedment. Sections were cut, collected on metal grids and stained with lead citrate before imaging. Further details can be found in the supplementary material. Mitochondrial ROS production was analyzed by FACS imaging (Amnis ImageStreamX Mk II, Cytek, Amsterdam, Netherlands). 2 × 10^6^ cells were seeded and treated with zVAD.fmk (20 μM) and S63845 (300 nM) and incubated for 20 h. Afterwards cells were stained with 500 nM MitoSOX red (ThermoFisher), washed and resuspended in PBS containing 2% FCS. 10,000 cells which were identified as single cells and in focus were measured and used for analyses. Pictures were obtained with 60× magnification. Data were analyzed with IDEAS® Image Analysis Software.

Additional Methods are available in the supplementary material.

## Results

### BH3-mimetics induce CICD in a subset of DLBCL cell lines

**Fig. 1:**
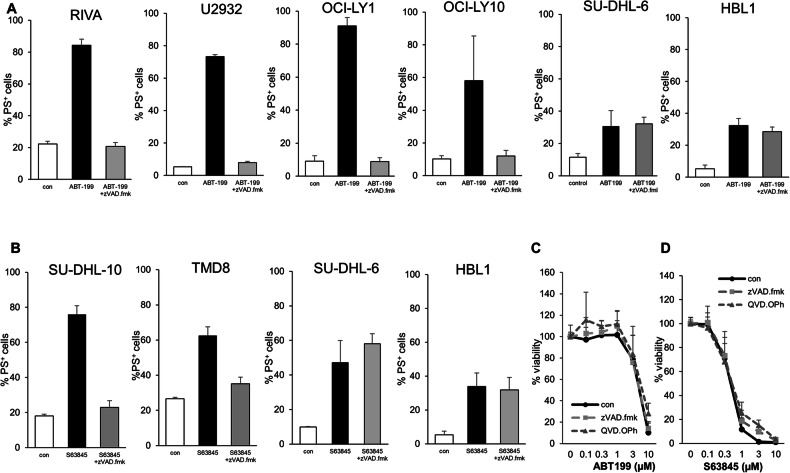
BH3-mimetics can trigger CICD in DLBCL cells. **A** DLBCL cell lines were exposed to ABT199 (RIVA: 10 nM, U2932: 100 nM, OCI-LY1: 300 nM, OCI-LY10: 10 μM, SU-DHL-6: 3 μM, HBL1: 1 μM) with or without 50 μM of zVAD.fmk for 24 h before analysis of cell death using staining of phosphatidylserine (PS) with AnnexinV-FITC and flow cytometry. **B** DLBCL cell lines were exposed to S63845 (SUDHL10: 100 nM, TMD8: 300 nM, SU-DHL-6: 300 nM, HBL1: 1 μM) with or without 50 μM of zVAD.fmk for 24 h before analysis of cell death using staining of phosphatidylserine (PS) with AnnexinV-FITC and flow cytometry. SU-DHL-6 cells were exposed to different concentrations of ABT199 (**C**) or S63845 (**D**) either alone or in the presence of zVAD.fmk (50 μM) or QVD.OPh (10 μM) for 72 h before analysis of viability using CTG analysis. Data shown are mean + S.D. of three independent experiments.

**Fig. 2:**
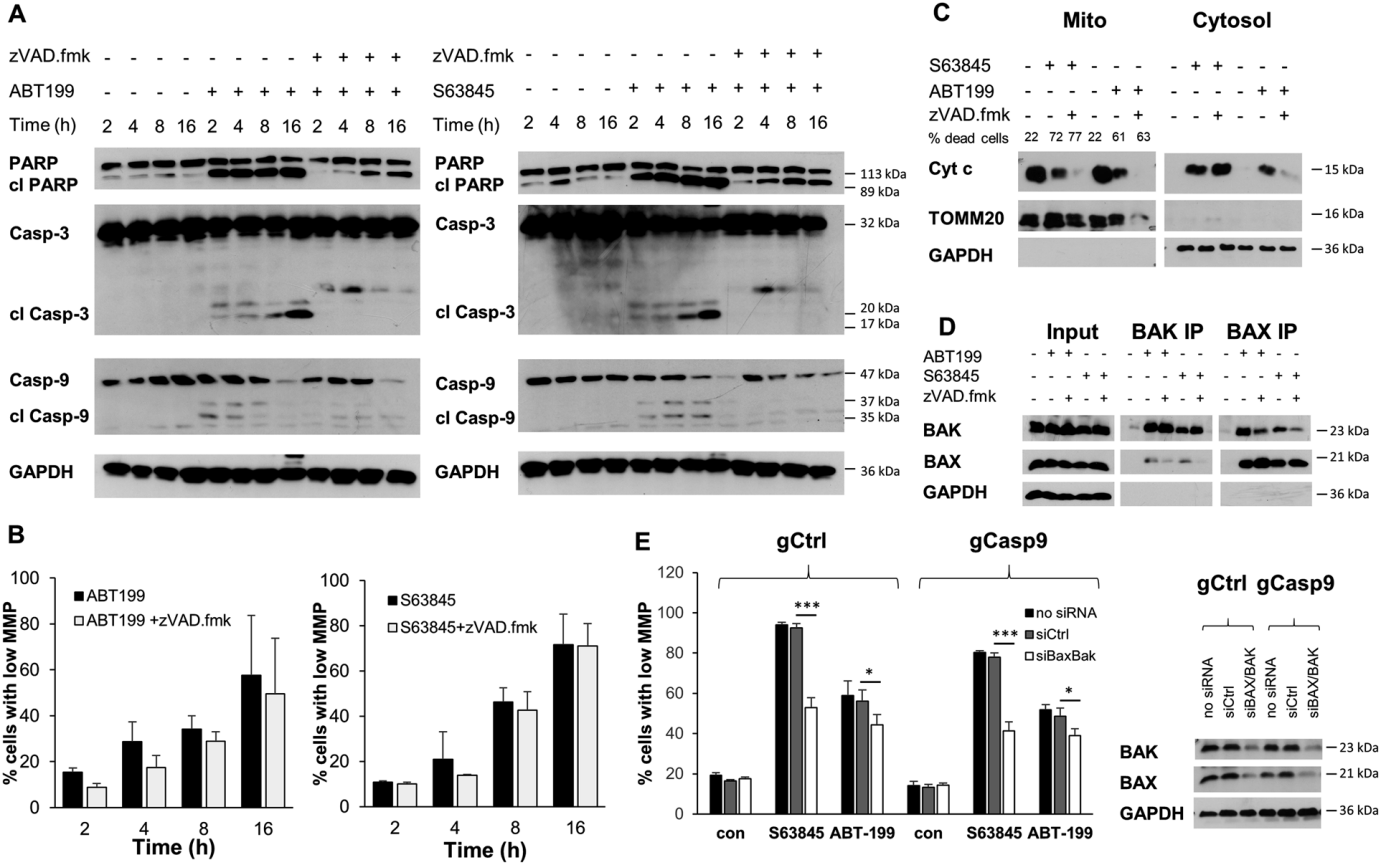
CICD involves permeabilization of the mitochondria by BH3-mimetics. **A** SU-DHL-6 cells were treated with ABT199 (3 μM, left) or S63845 (300 nM, right) with or without zVAD.fmk (20 μM) for the indicated time points before protein lysis and analysis of caspase or PARP cleavage by Western blotting. **B** SU-DHL-6 cells were treated with ABT199 (3 μM, left) or S63845 (300 nM, right) with or without zVAD.fmk (20 μM) for the indicated time points before analysis of MMP using staining with TMRM and flow cytometry. Data shown are mean + S.D. of three independent experiments. **C** SU-DHL-6 cells were treated with ABT199 (3 μM) or S63845 (300 nM) with or without zVAD.fmk (20 μM) for 20 h before differential lysis and analysis of cytochrome c release from mitochondrial into cytosol by Western blotting. **D** SU-DHL-6 cells were treated with ABT199 (3 μM) or S63845 (300 nM) with or without zVAD.fmk (20 μM) for 20 h before analysis of BAX and BAK activation using IP of active BAX/BAK with conformation-specific antibodies. **E** SU-DHL-6 cells with CRISPR/Cas9-mediated deletion of caspase-9 (gCasp9) or corresponding control cells (gCtrl) were transfected with BAX/BAK or control siRNA before treatment with S63845 (300 nM, 16 h) or ABT199 (3 μM, 20 h) and analysis of MMP using staining with TMRM and flow cytometry (data shown are mean + S.D. (*n* = 3), ***p < 0.001; *p < 0.05). Knockdown efficiency was assessed by Western blotting with GAPDH serving as loading control.

To investigate whether BH3-mimetics can induce CICD in DLBCL, we screened different DLBCL cell lines sensitive to the BCL2 inhibitor ABT199, the BCL-X_L_ inhibitor A1331852 or the MCL1 inhibitor S63845 [8] in the presence of the broad range caspase-inhibitor zVAD.fmk. Whilst most (8/10) cell lines displayed mainly classical caspase-dependent apoptosis, some cell lines, including SU-DHL-6 and HBL1 cells, also underwent cell death after addition of zVAD.fmk, suggesting the induction of CICD. As the SU-DHL-6 cells are a Germinal-Center B-cell (GC) type of DLBCL, and the HBL1 an Activated B-cell (ABC) type, the occurrence of CICD appeared to not be restricted to one of the main subtypes of DLBCL. Of note, CICD was also independent of the BH3-mimetic used, since both ABT199 and S63845 induced CICD in SU-DHL-6 and HBL1 cells (Fig. 1A, B, Supplementary Fig. 1). To exclude the possibility that this phenotype was zVAD.fmk-dependent, we tested another caspase inhibitor (QVD.OPh), which showed the same effect (Fig. 1C, D). Analysis of caspase cleavage indicated that the addition of zVAD.fmk blocked caspase-3 activation as well as cleavage of the caspase target PARP, confirming that caspase activity was inhibited by zVAD.fmk (Fig. 2A and Supplementary Fig. 2A). To exclude that the observed CICD is an artifact induced by caspase inhibitors we extended our studies to a genetic model where intrinsic apoptosis was inhibited by CRISPR/Cas9-mediated deletion of caspase-9 (Supplementary Fig. 2B). SU-DHL-6 cells with deletion of caspase-9 still underwent BH3-mimetic-induced cell death, confirming that BH3-mimetics can trigger CICD (Supplementary Fig. 2C).

### Upstream mitochondrial apoptotic signaling is induced independently of caspases

To investigate how cell death signaling was affected when caspases were blocked, we examined typical signaling events occurring during intrinsic apoptosis. First, we asked whether the kinetics of MOMP differed between CICD and apoptosis. Analysis of the MMP by staining with TMRM indicated that MOMP was induced at similar kinetics during apoptosis and CICD, with loss of MMP occurring rapidly within 4–8 h of treatment with BH3-mimetics irrespective of caspase activity (Fig. 2B). To analyze the release of cytochrome *c*, cells were separated into heavy membrane (HM) fractions containing mitochondria, and cytosolic fractions containing soluble proteins. Release of cytochrome *c* was induced by BH3-mimetics irrespective of caspase activation. Interestingly, the levels of cytochrome *c* in the mitochondria were reduced more dramatically in CICD conditions, although not consistently more cytochrome c was released into the cytosol (Fig. 2C). Similarly, TOMM20 levels were reduced during CICD, suggesting a loss of mitochondrial proteins. Next, we examined the activation of BAX and BAK using conformation-specific antibodies and immunoprecipitation (IP). While both BAX and BAK changed conformation during apoptosis or CICD, the formation of heterodimers appeared to be reduced under CICD conditions as compared to apoptosis (Fig. 2D). To investigate the function of BAX and BAK during apoptosis and CICD, we performed siRNA-mediated silencing of BAX and BAK in the caspase-9 deleted or control cells. In line with their prominent role in mitochondrial perturbations, silencing of BAX and BAK resulted in reduced loss of MMP upon treatment with BH3-mimetics, which was observed both in control cells and in caspase-9 deleted cells (Fig. 2E). Taken together, these data demonstrate that the kinetics of BAK/BAX activation and its effects on MOMP and cytochrome *c* release are mainly independent of caspase activity.

### Mitochondrial morphology differs between apoptosis and CICD

**Fig. 3:**
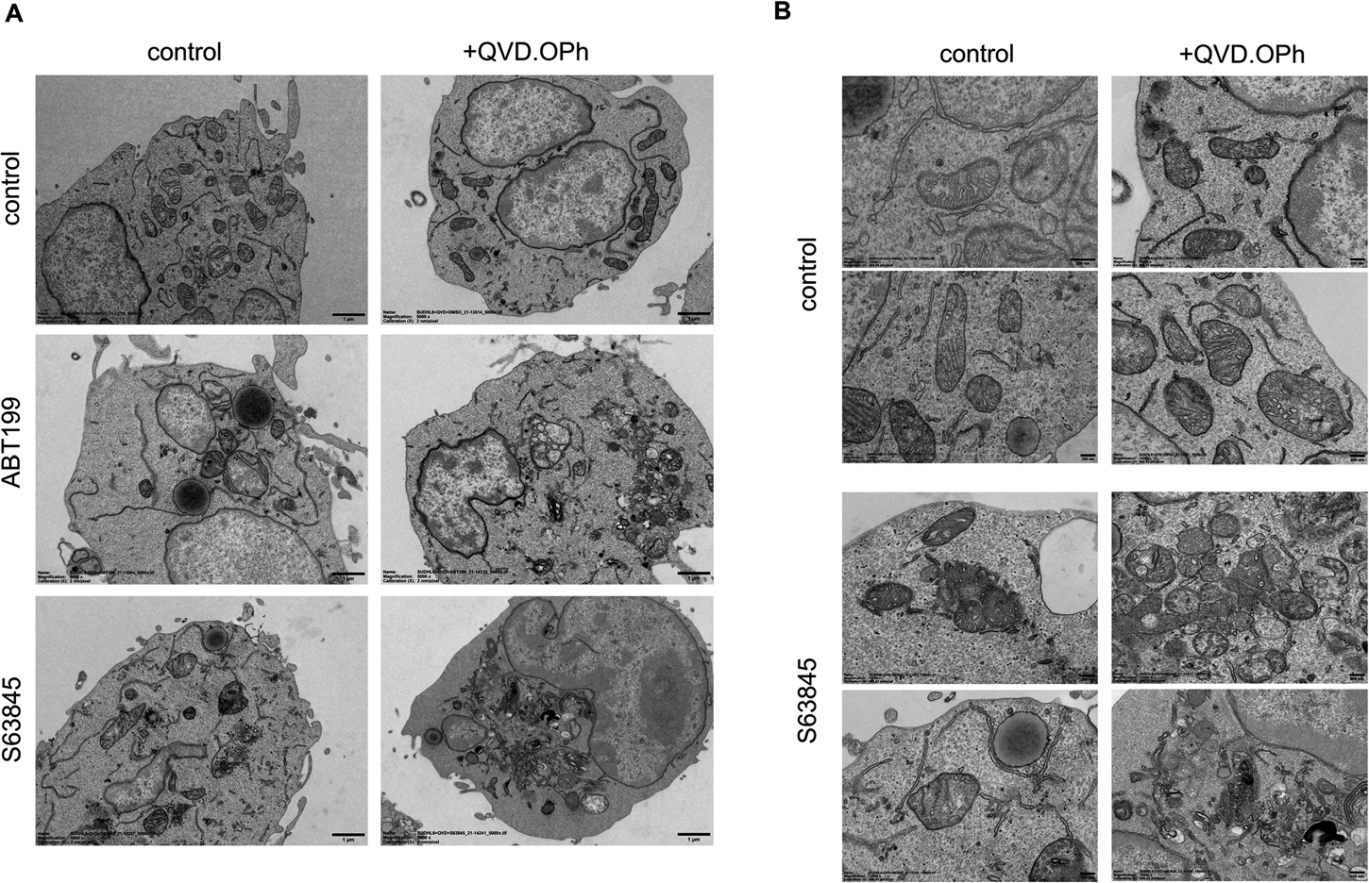
CICD following BH3-mimetics in DLBCL occurs with altered mitochondrial phenotypes. **A** SU-DHL-6 cells treated with ABT199 (3 μM for 16 h) or S63845 (300 nM for 8 h, right) with or without QVD.OPh (10 μM) were analyzed by electron microscopy (Scale bar: 1 μm, Magnification: 5000×). **B** Higher magnification displays diverse mitochondrial morphology upon treatment with S63845 (300 nM) in the presence or absence of QVD.OPh (10 μM) for 16 h. Two representative images are shown for each condition (Scale bar: 200 nm, Magnification: 25,000×).

Next, we asked how the mitochondrial morphology was affected during apoptosis and CICD conditions. We had previously shown that the anti-apoptotic BCL2 proteins are important for maintaining mitochondrial integrity, and that under apoptotic conditions, BH3-mimetics may induce severe mitochondrial perturbations including swelling, decreased matrix density and loss of cristae structures in malignant B-cells [23, 24]. To analyze how the mitochondrial morphology differed during CICD, we performed electron microscopy studies on SU-DHL-6 cells following treatment with BH3-mimetics with and without QVD.OPh. While control cells appeared healthy with normal chromatin morphology and large mitochondria with regular cristae morphology, treatment with BH3-mimetics induced severe mitochondrial perturbations including swelling as well as condensation of mitochondria. Notably, mitochondria were even more damaged during CICD as compared to apoptosis, with only few recognizable mitochondria with intact cristae formation remaining. In particular treatment with S63845 and QVD.OPh resulted in condensed mitochondria with signs of fission or mitochondrial fragmentation and an accumulation of small degradative compartments or vesicles resembling autophagosomes and lysosomes, suggesting increased degradation of mitochondria (Fig. 3). Further evidence for a loss of mitochondria was provided by loss of mitochondrial staining and increased LC3-I to LC3-II processing, which was observed during CICD but not during apoptosis (Supplementary Fig. 3).

### BH3-mimetics do not induce necroptosis or ferroptosis

**Fig. 4:**
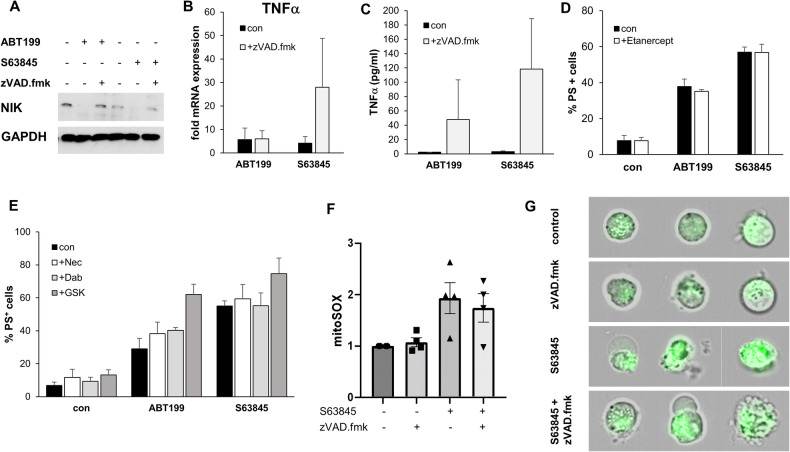
CICD coincides with increased TNFα production but does not involve necroptosis. **A**–**C** SU-DHL-6 cells were treated with ABT199 (3 μM) or S63845 (300 nM) with or without zVAD.fmk (20 μM) for 24 h (ABT199) or 16 h (S63845). A) After protein lysis NIK expression was analyzed by Western blotting. **B** mRNA expression of TNFα was analyzed by qRT-PCR and expressed as fold change of untreated control cells. **C** Release of TNFα into the culture supernatant was quantified by Chemokine Bead Array. **D** The influence of secreted TNFα on cell death was analyzed by blocking of TNFα with etanercept followed by analysis of cell death using staining of PS with AnnexinV-FITC and flow cytometry. **E** The role of necroptosis was analyzed by addition of Nec-1s (Nec, 30 μM) or Dabrafenib (Dab, 5 μM) or GSK872 (15 μM) followed by analysis of cell death using staining of PS with AnnexinV-FITC and flow cytometry. **F**, **G** SU-DHL-6 cells were treated with S63845 (300 nM) with or without zVAD.fmk (20 μM) for 16 h (S63845) before staining with MitoSOX and ImageStream analysis. **F** Normalized MitoSOX fluorescence is presented with untreated cells set as 1. Data shown are mean + S.D. of four independent experiments with 10.000 cells analyzed per condition. **G** Example images are shown for each condition as overlay of transmitted light and MitoSOX fluorescence.

A previous report has shown the induction of necroptosis by BH3-mimetics, which is mediated by an accumulation of the NF-kB regulator NIK followed by nuclear translocation of p65, TNFα synthesis, IAP downregulation and subsequently necroptosis [15]. Here, we observed a loss of NIK under apoptotic conditions, whereas NIK was unaffected under CICD conditions (Fig. 4A). In addition, we observed no change in the expression of cIAP1/cIAP2 and a reduced phosphorylation of RIPK1 upon treatment with BH3-mimetics (Supplementary Fig. 4). Interestingly, TNFα mRNA was induced, which also resulted in increased release of TNFα into the medium during CICD (Fig. 4B, C). However, blocking of TNFα using etanercept did not affect CICD (Fig. 4D). Furthermore, blocking of RIPK1 or RIPK3 with Necrostatin-1s (Nec-1s), Dabrafenib or GSK872 did not prevent CICD (Fig. 4E), effectively eliminating necroptosis induction as a potential pathway during CICD in this setting.

Next, we asked whether ROS accumulated under CICD conditions. ROS were induced by treatment with BH3-mimetics, as indicated by increased H2DCF and MitoSOX staining (Supplementary Fig. 5A). However, ROS induction was higher during apoptosis than CICD, which was confirmed by ImageStream analysis (Fig. 4F, G). Here, analysis at a single cell level showed increased ROS formation under both apoptotic and CICD conditions, with higher mitoSOX staining observed under apoptotic conditions (Fig. 4G). To investigate whether ROS or the release of mtDNA associated with mitochondrial damage may activate the cGAS/STING pathway, we analyzed the expression of P-TBK and P-IRF3 and found no indication of activated cGAS/STING signaling neither under either apoptotic or under CICD conditions (Supplementary Fig. 5B). The innate immune sensor Z-DNA Binding Protein 1 (ZBP1) was not expressed on mRNA level and also not induced during either apoptosis or CICD (Supplementary Table 1). Since ROS are also involved in ferroptosis, we next asked whether cell death may be inhibited by the inhibitors liproxstatin-1 or ferrostatin-1. While cell death induced by the positive control Erastin was blocked by the addition of either ferrostatin-1 or liproxstatin-1, cell death induced by BH3-mimetics and zVAD.fmk was not inhibited (Supplementary Fig. 5C), demonstrating that ferroptosis was not the main route of CICD observed here.

### CICD occurs with global changes in mRNA expression associated with JNK/AP1 induction and modulation of the immune response

**Fig. 5:**
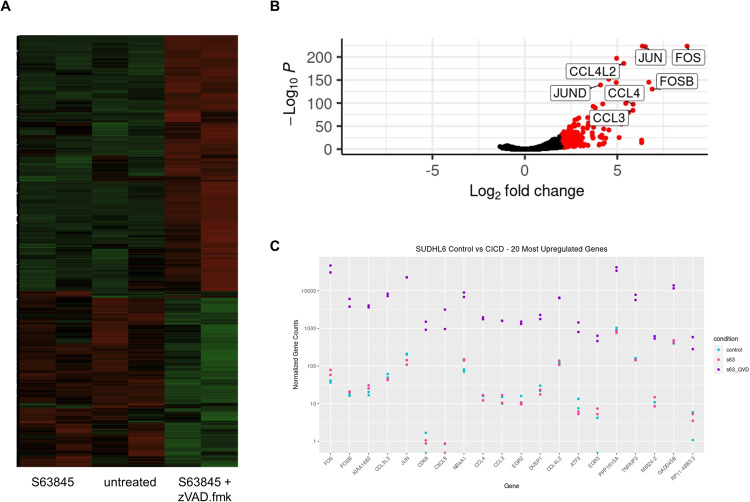
CICD is associated with transcriptional changes. **A**–**C** SU-DHL-6 cells were treated with S63845 (300 nM) with or without zVAD.fmk (20 μM) for 8 h before isolation of RNA for RNA-Sequencing (*n* = 2). **A** Heatmap showing clustering of S63845 with the untreated control sample and transcriptional changes under CICD conditions. **B** Volcano blot showing upregulation of genes under CICD conditions. Differentially expressed genes (DEGs) were determined using *p* values (*p* < 0.05). **C** The most significantly upregulated genes are shown with the normalized gene count. For full list see Supplementary information.

To obtain detailed insights into the signaling events during CICD we next performed global RNA sequencing (Supplementary Table 1). While apoptotic cells displayed little transcriptional changes at the early time point (8 h) chosen for this analysis, induction of CICD by S63845 and zVAD.fmk was associated with striking transcriptional changes, which differed from the transcriptional profile in untreated or apoptotic cells by unsupervised clustering (Fig. 5A).

**Fig. 6:**
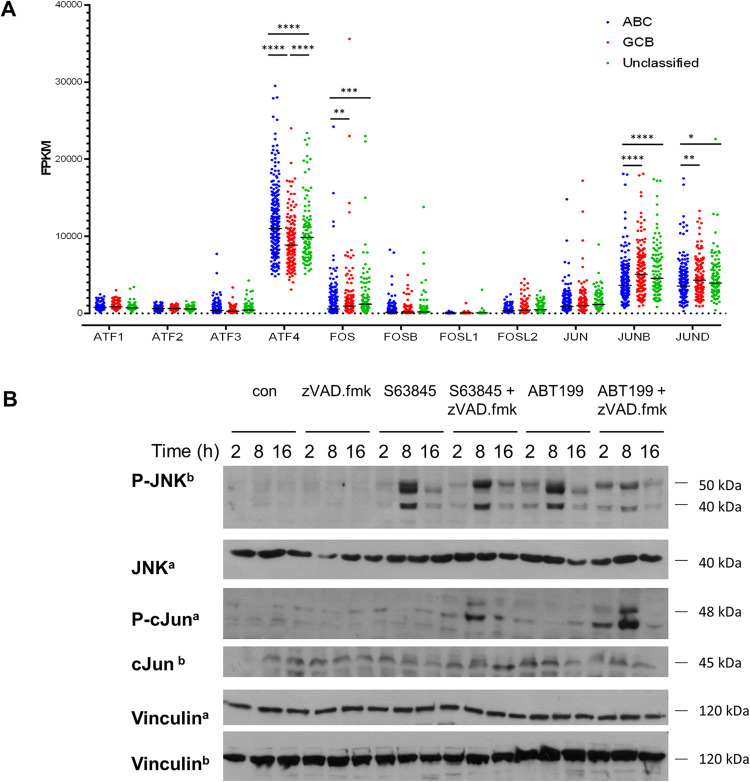
Activation of AP1. **A** Expression of the AP1 family genes was analyzed in a database containing 574 DLBCL patients [39]. Patients were classified according to their main subtype and gene expression was presented as fragments per kilobase million (fpkm). ****p < 0.0001; ***p < 0.001; **p > 0.01; *p < 0.05). **B** SU-DHL-6 cells were treated with S63845 (300 nM), ABT199 (1 μM) with or without zVAD.fmk (20 μM) for 2, 8, or 16 h before protein purification and Western blotting with Vinculin serving as loading control. Please note that the same samples were analyzed on two separate gels and a loading control is shown for each gel. Representative blots of 4 independent biological repeats are shown.

A comparison of untreated cells and cells undergoing CICD revealed that many genes were significantly down- (*n* = 1311) or upregulated (*n* = 2162) (Fig. 5B). Amongst the 20 most significantly upregulated genes were several members of the AP1 transcription factor family (JUN, JUND, FOS, FOSB) with FOS and JUN being the two most significantly upregulated genes (*p* = 10^−274^ and *p* = 10^−260^). All other AP1 family members (JUND, FOSL1, FOSL2, ATF1, ATF2, ATF3, ATF4) were also all significantly upregulated during CICD. In addition, IL8 (encoded by CXCL8) and several chemokines were highly significantly upregulated, including CCL3 and CCL4 (Fig. 5C). Confirmation by qRT-PCR showed similar expression changes, thus validating the global transcriptome analysis (Supplementary Fig. 6). These data indicate a prominent role of AP1 during CICD. Analysis of a publicly available patient cohort revealed high mRNA expression of the AP1 transcription factors across the different subtypes of DLBCL, with particularly high expression of ATF4, FOS, JUN, JUNB and JUND (Fig. 6A).

The main upstream inducer of the AP1 transcription factors is the JNK pathway, leading to phosphorylation and enhanced transcriptional activity of cJun. To investigate whether the JNK pathway is activated during CICD we performed detailed kinetic analysis upon treatment with BH3-mimetics. Treatment with either ABT199 or S63845 resulted in a time-dependent phosphorylation of JNK at 2 or 8 h of treatment, which was independent of caspase activity. However, this resulted in phosphorylation of cJun at 8 h of treatment only when caspase activation was prevented (Fig. 6B), suggesting that during apoptosis and the associated caspase activation, JNK-mediated activation of AP1 was blocked.

**Fig. 7:**
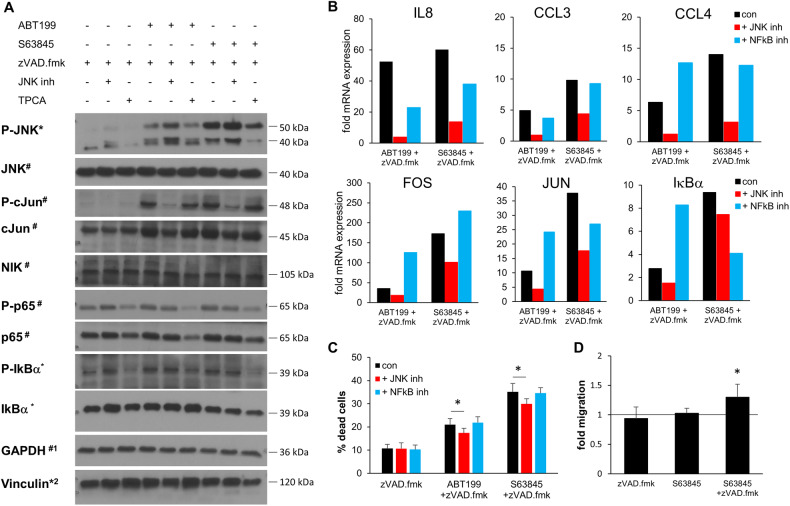
CICD is mediated by JNK activation. SU-DHL-6 cells were treated with ABT199 (3 μM) or S63845 (300 nM) with or without zVAD.fmk (20 μM) and inhibitors for JNK (JNK Inh VIII, 3 μM) or NFkB (TPCA-1, 1 μM) for 8 h. **A** Protein expression was analyzed by Western blotting. **B** mRNA expression was analyzed by qRT-PCR and expressed as fold change of untreated control cells. **C** Cell death was analyzed by FSC/SSC analysis and flow cytometry. Data shown are mean + S.D. (*n* = 3). *p < 0.05. **D** Conditioned medium supernatant of SU-DHL-6 cells treated with S63845 (300 nM) with or without zVAD.fmk (20 μM) for 16 h was used as chemoattractant in a transwell migration assay. Migration of IL15-activated NK cells were analyzed by flow cytometry using counting beads. Data shown are mean + S.D. of three different NK cell donors and two different conditioned mediums relative to untreated control medium (*p < 0.05).

Since FOS and JUN are transcriptional targets of both the AP1 and the NF-kB signaling pathway, we investigated the functional importance of these main signaling pathways by including specific inhibitors for JNK and for IKK-2. Increased phosphorylation of cJun during CICD was inhibited by the JNK inhibitor but not the IKK inhibitor (Fig. 7A). Components of the NF-κB pathway were also investigated, but neither phosphorylation of p65 nor of IkBα was altered during CICD. In line with the observations on protein expression, addition of the JNK inhibitor prevented the mRNA upregulation of IL8, CCL3 and CCL4 during CICD, while the IKK inhibitor had little effect (Fig. 7B). To investigate whether JNK-mediated AP1 activation was contributing to CICD we analyzed cell death in the presence of the JNK or IKK inhibitor. These experiments revealed a small but significant reduction in CICD upon JNK inhibition, but not upon IKK inhibition, suggesting that the JNK-mediated AP1 activation contributed to CICD (Fig. 7C). Finally, we asked whether the induction of CICD and the coinciding upregulation of pro-inflammatory chemokines may affect neighboring immune cells and alter the immune response towards the dying cells. To this end, we performed a transwell migration assay with IL15-activated NK cells using the supernatant of SU-DHL-6 cells treated with BH3-mimetics plus or minus zVAD.fmk as chemoattractant. Interestingly, NK cells migrated significantly more towards the CICD supernatant, indicating that the induction of CICD may increase the immune response towards the dying cells (Fig. 7D).

## Discussion

By selectively neutralizing the anti-apoptotic BCL2 proteins, BH3-mimetics are prototypic inducers of apoptosis. Besides apoptosis, also several other forms of cell death have been shown to originate from mitochondria, including necroptosis or mitophagy, that can both occur in the absence of active caspases. Here, we describe an entirely novel form of CICD that, to the best of our knowledge, is distinct from any other form of CICD described before and that occurs in a subset of DLBCL cells treated with BH3-mimetics.

MOMP has been established as a point of no return that ensures cell death as a failsafe mechanism in case apoptosis execution should be prevented [25]. Thereby, MOMP can either directly cause CICD or, alternatively, cells die due to loss of mitochondrial function and respiratory shutdown. The latter however takes longer and is usually associated with delayed cell death occurring at days rather than hours after MOMP, with cells completely devoid of mitochondria being able to survive for several days before dying [26]. Here, we observe rapid CICD occurring within hours of treatment and at similar kinetics as apoptosis. Therefore, we hypothesize that CICD is an active form of cell death rather than a consequence of mitochondrial impairment. Active forms of CICD may be caused by the mitochondrial release of toxic components such as mtDNA, AIF or Smac. By binding to IAPs and triggering their proteasomal degradation, released Smac may contribute to necroptosis. In line with this hypothesis, the BH3-mimetic ABT-737 has been shown to induce CICD in endothelial SVEC cells upon caspase inhibition [15]. Mechanistically, the authors observed IAP degradation, NIK accumulation and NF-κB-dependent induction of necroptosis via release of TNFα, which was blocked by deletion of NIK. Here, we did not observe IAP degradation or NIK accumulation, and addition of eternacept to block TNFα did not inhibit CICD. Moreover, in contrast to our study, CICD in SVEC cells occurred at significantly slower kinetics compared to apoptosis, highlighting that in our study CICD was induced by a different pathway as observed by Giampazolias et al. [15].

The BCL2 protein family has several links to mitophagy, and BH3-mimetics have been shown to promote mitophagy e.g. by directly displacing the BH3 domain-containing protein Beclin-1 from BCL2 or BCL-X_L_ [22, 27]. In this regard, the induction of mitophagy may either represent a survival mechanism or lead to cell death [28, 29]. Several observations in our study support the induction of mitophagy selectively during CICD but not during apoptotic cell death, including the ultrastructural analysis, the accumulation of LC3-II associated with increased autophagosome formation, and lastly the loss of mitochondrial mass suggested by staining with MitospyGreen and loss of cytochrome c and TOMM20. Taken together, these data suggest the induction of mitophagy by BH3-mimetics under conditions when caspase-driven apoptosis is blocked. Of note, this phenotype was induced by either ABT199 (targeting BCL2) or by S63845 (targeting MCL1), indicating that this is not restricted to the function of a specific BCL2 protein, but rather a result of MOMP. In this context, a dependence upon autophagy associated with a transient decrease in mitochondrial mass has previously been observed during CICD as a pro-survival mechanism [30]. In that study, autophagy induced by GAPDH, increased ATP production and high Atg12 expression protected cells from CICD.

**Fig. 8:**
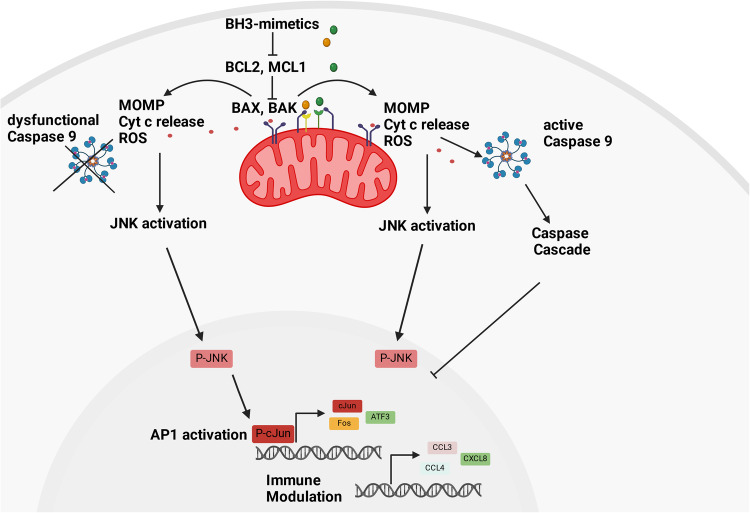
Schematic representation of the signaling pathways associated with CICD or apoptosis. By binding to their anti-apoptotic targets, BH3-mimetics trigger the activation of BAX and BAK, leading to MOMP and release of cytochrome c into cytosol. During apoptosis, this results in formation of the apoptosome complex, activation of caspase-9 and caspase cascade. Upon inhibition of caspase-9, JNK activation is induced, which leads to activation of the AP1 transcription factor family, a pathway that is blocked during apoptosis. Transcriptional reprogramming by AP1 involves release of pro-inflammatory cytokines and modulation of the immune response during CICD, but not during apoptosis. Figure was created with Biorender.com.

In contrast to the other forms of CICD described previously, in our study CICD is associated with rapid and prominent transcriptional changes that were only observed in cells undergoing CICD but not apoptosis. In particular, CICD occurred with an upregulation of multiple AP1 family transcription factors including JUN and FOS. Inhibition of upstream JNK signaling revealed that the upregulation of AP1 and associated transcriptional changes were mediated by JNK but not by NF-κB signaling. These observations highlight a novel function of JNK signaling in mediating CICD rather than apoptosis (Fig. 8). However, these results also open up new questions regarding the signaling events underlying CICD. To this end, it is currently unclear how MOMP induced by BH3-mimetics activates JNK signaling. Common inducers of JNK signaling are ROS [31], and increased ROS were observed both under apoptotic and under CICD conditions, with more ROS being produced during apoptosis. Since the transcriptional changes following JNK activation are only observed when caspases are blocked, one hypothesis could be that caspases somehow shut down the CICD signaling pathway and the transcriptional reprogramming associated with it. Other open questions are why only a subset of lymphoma cells appears to be able to activate this CICD pathway, and how relevant the route of cell death is in the context of the microenvironment and response to treatment in vivo. In line with the study by Giampazolias et al. [15], also in the DLBCL cells studied here, the induction of CICD following MOMP may have profound effects on the immune response. Besides TNFα, also IL-8, CCL3 and CCL4 are induced following JNK activation during CICD, some of which have been described as AP1 transcriptional targets [32] and serve as important chemoattractants for immune cells. Of note, in a subset of DLBCL patients, the infiltration of neutrophils following IL8 expression has been implicated in tumor progression, possibly due to production of the proliferation-inducing ligand APRIL by the neutrophils [33, 34]. In addition, IL8 and IL6 have been shown to be increased in cells during chronic exposure to BH3-mimetics [3]. Here, we were able to show that the induction of CICD but not of apoptosis increases the migration of NK cells towards the dying cells. This could be highly relevant for the immunosurveillance of lymphoma, since NK cells have the capacity to attack lymphoma cells, but are often inhibited and reduced in DLBCL patients [35, 36]. Therefore, the induction of CICD with the coinciding release of pro-migratory chemokines may positively influence the NK cell-mediated attack of DLBCL cells and may be highly relevant in particular for combination treatment with antibodies like rituximab or obinutuzumab, which act by inducing antibody-dependent cytotoxicity and hence require NK cells to be attracted to the lymphoma cells [37, 38]. Although the relevance of CICD for the treatment of lymphoma patients remains to be investigated, the high expression of several AP1 transcription factors in DLBCL patient tissues across the different subtypes supports a potential role of AP1 in cell death induced by mitochondrial perturbations.

Taken together, our study describes a novel mode of CICD induced by BH3-mimetics in a subset of DLBCL cell lines that is associated with JNK activation, AP1-mediated transcriptional reprogramming, the release of pro-inflammatory cytokines and increased migration of NK cells.

### Supplementary information

Original Data File

Original Data File

Original Data

## Data Availability

RNA Sequencing data are available at GEO (GSE263183). All other data are available at the corresponding author at reasonable request.
