# Supplementary material for: Identification of a novel form of caspase-independent cell death triggered by BH3-mimetics in diffuse large B-cell lymphoma cell lines
Source: Cell Death Dis. 2024 Apr 15;15(4):266. doi: 10.1038/s41419-024-06652-3 (PMC11018778; doi:10.1038/s41419-024-06652-3)

Supplementary Material and Methods

Chemicals

Cells were treated with Dabrafenib (Selleck Chemicals), Nec1s (Merck, Darmstadt, Germany), Etanercept (Merck), TPCA-1 (Sigma-Aldrich, St. Louis, MS), JNK-INH VII (Biomol GmbH, Hamburg, Germany), Bafilomycin A (Selleck), Erastin (Sigma-Aldrich), Ferrostatin-1 (Sigma-Aldrich) or Liproxstatin-1 (Sigma-Aldrich).

Cell culture and CRISPR-Cas9-mediated knockout (KO)

DLBCL cell lines were obtained from the Leibniz Institut Deutsche Sammlung von Mikroorganismen und Zellkulturen DSMZ (Braunschweig, Germany) and routinely tested for mycoplasma contamination. Identity of cell lines was confirmed by STR profiling performed by DSMZ. Cells were cultured in RPMI-1640 GlutaMAX-I (Life Technologies, Eggenstein, Germany) supplemented with 10 % fetal calf serum (Life Technologies) and 1 % penicillin/streptavidin (Life Technologies). To delete caspase-9, SUDHL6 cells were transduced with pCW-Cas9-Blast (#83481, Addgene, Watertown, MA) and a stable Cas9-inducible clone was selected. These cells were transduced with Lentiguide-Puro (#52963, Addgene) containing a control gRNA sequence or three different caspase-9 targeting sequences (ACACCCAGACCAGTGGACAT, CAATCTTCTCGACCGACACA, TGGATGTCCTCGATCATATG). After induction of Cas9 using doxycycline (Merck) and selection with puromycin (Thermo Scientific, Waltham, MA), stable clones were expanded and analyzed for caspase-9 deletion by Western blotting. Silencing of BAX and BAK was performed with the following Silencer Select siRNAs (Thermo Fisher Scientific): BAX (s1888, s1890) and BAK (s1880, s1881). Cells were electroporated twice with 50 nM of siRNA using P14 on Neon transfection device (Invitrogen). Treatment with BH3-mimetics was started 6 hours after the second transfection.

NK cells were isolated from peripheral blood mononuclear cells (PBMCs) derived from healthy donors (DRK Blutspendedienst, Frankfurt, Germany) by density gradient centrifugation with

Histopaque-1077 (Sigma-Aldrich) and enriched with Human NK Cell Enrichment kit (STEMCELL Technologies, Cologne, Germany). To expand and activate NK cells, isolated NK cells were cultured for 18 days in NK-MACS medium (Miltenyi Biotec, Bergisch Gladbach, Germany) supplemented with 10 ng/ml IL-15 (Peprotech, Rocky Hill, CT). Purity and activation of NK cells were routinely controlled by staining of CD56/CD16 and flow cytometry at FACS Canto II (BD Biosciences, Heidelberg, Germany) as described previously (1).

BAX/BAK activation immunoprecipitation (IP)

For detection of active BAX or BAK, cells were lysed in CHAPS lysis buffer (10 mM HEPES (pH 7.4); 150 mM NaCl; 1% CHAPS). IP was performed by incubation of protein lysate overnight at 4°C with 2 µg/ml mouse anti-BAX antibody (6A7, Sigma-Aldrich) or anti-BAK antibody (Ab-1; Merck) and 10 µl pan-mouse IgG Dynabeads (Dako, Hamburg, Germany) followed by washing with CHAPS buffer. The precipitate was analyzed for BAX and BAK expression by Western blotting using the BAX NT antibody (#ABC11, Merck) or BAK antibody (#556393, BD Biosciences).

Protein lysis, fractionation and Western blotting

For standard whole cell lysates, cells were lysed in TritonX-containing Tris buffer for 30 minutes. For fractionation, cells were permeabilized in 0.025 % digitonin for 10 minutes followed by centrifugation at 13 000 rpm for 3 minutes. Supernatant-containing soluble proteins was classified as “cytosol”, whereas the pellet containing heavy membrane (HM) fractions were classified “mitochondria”. Western blotting was performed using the following primary antibodies: P-JNK (#44-682G, Thermo Scientific), JNK (#44-690G, Thermo Scientific), Casp9 (#9502, Cell Signaling, Danvers, MA), PARP (#9542, Cell Signaling), Casp3 (#9662, Cell Signaling), LC3 (#PA1-16930, Thermo Scientific), GAPDH (#5G4cc, Biotrend), Vinculin (#V9131, Merck), NIK (#4994, Cell Signaling), P-cJun (#2361, Cell Signaling), cJun (#2315, Cell Signaling), P-p65 (#3033, Cell Signaling), p65 (sc8008, Santa Cruz Biotechnology, Santa

Cruz, CA), P-IkBa (#9246, Cell Signaling), IkBa (#9242, Cell Signaling), PINK1 (#6946, Cell Signaling), BNIP3L (#12396, Cell Signaling), Cytc (#556433, BD Biosciences), RIP1 cIAP1 cIAP2. For detection, goat anti-rabbit or goat anti-mouse IgG conjugated to horseradish peroxidase (#SC-2004, SC-2005, Santa Cruz) and enhanced chemiluminescence (Amersham Biosciences, Freiburg, Germany) were used.

RNA isolation, qPCR, RNA sequencing and data analysis

Total cellular RNA was isolated using the peqGOLD MicroSpin total RNA kit and peqGOLD total RNA kit (PeqLab, Erlangen, Germany). For cDNA synthesis 1 µg of total RNA was used for RevertAid first strand cDNA synthesis kit (ThermoFisher, Roskilde, Denmark). qRT-PCR was performed on a QuantStudio™ 7 Flex system (Applied Biosystems, Darmstadt, Germany) using Sybr™ Green PCR master mix (Applied Biosystems) and following primers: TNFα (ACAACCCTCAGACGCCACAT, TCCTTTCCAGGGGAGAGAGG), CCL3 (CTTAGCCCCCTAGTCTCCAGT, GTCACACGCATGTTCCCAAG), CCL4 (GCTTCCTCGCAACTTTGTGG, TCACTGGGATCAGCACAGAC), IL-8 (CTCTTGGCAGCCTTCCTGATT, TATGCACTGACATCTAAGTTCTTTAGCA), FOS (AACCGCCACGATGATGTTCT, GTGAGCTGCCAGGATGAACT), c-JUN (GTCCGAGAGCGGACCTTATG, CTTTTTCGGCACTTGGAGGC), IkBa (GTCAAGGAGCTGCAGGAGAT, ATGGCCAAGTGCAGGAAC) G6PDH (ATCGACCACTACCTGGGCAA, TTCTGCATCACGTCCCGGA), RPII (GCACCACGTCCAATGACAT, GTGCGGCTGCTTCCATAA). Human mRNA sequencing was performed using Illumina PE150 platform at Novogene (Cambridge, UK). Fastq files were aligned to human reference genome GRCh38 using STAR. Generated BAM files were sorted and indexed using Samtools v1.9. RSubread was used to quantify gene abundance. Differential gene expression analysis was performed using DESeq2 (with a target cut-off of padj<0.05 and L2FC <-1.5 or >1.5). Plots were produced using R packages ggplot, complex heatmap, and enhancedVolcano. The ALICE High Performance Computing Facility at the University of Leicester was used for bioinformatic analysis. Gene counts are shown in

Supplementary Table 1. For expression of AP1 family genes in DLBCL a publicly available database (2) was interrogated and gene expression was analysed in GraphPad Prism.

Migration assay

To investigate the effects of apoptosis or CICD on the migration of NK cells, SUDHL6 cells were exposed to S63845 (300 nM) for 16 hours in the presence or absence of 20 μM zVAD.fmk. Cells were removed by centrifugation and conditioned medium was filtered with a 0.22 μm mesh. Conditioned medium (500 μl) was used in a transwell migration chamber with 3 μm pore size (Corning, Corning, NY). Purified NK cells were cultured and activated for 2 weeks with IL-15 before migration was assessed in RPMI1640 medium without FCS and with 0.5 % bovine serum albumin. NK cells that migrated into the lower chamber were analyzed after 3 hours using flow cytometry with 5 μl of calibrating beads added per sample.

Electron Microscopy

Samples were fixed in 2.5% glutaraldehyde (Agar Scientific, UK) in 0.1M sodium cacodylate buffer (pH 7.4) at 4°C overnight before further fixation in 1% osmium tetroxide (Agar Scientific, UK) / 1.5% potassium ferricyanide (Sigma, UK) in buffer. Following a 45-minute incubation in the dark in 1% tannic acid in 0.05M sodium cacodylate, cells were placed in 1% sodium sulphate in 0.05M cacodylate for 5 minutes and washed in ddH2O. Pellets were embedded into 3% liquid agar, cut into 1mm3 pieces followed by dehydration steps in ethanol (30%, 50%, 70%, 90% and 100%). Samples were transferred through a series of propylene oxide (Sigma, UK) and 812 resin (TAAB Laboratories, UK), then embedded in 100% resin which was polymerized at 60°C for 24 hours. 70nm sections were cut using Reichert Ultracut E ultramicrotome, collected onto copper mesh grids and stained with lead citrate for 7 minutes. Grids were viewed on JEM-1400 TEM (JEOL Ltd, Welwyn Garden City, UK) at an accelerating voltage of 120 kV and digital images were collected with an Xarosa digital camera with Radius software (EMSIS, Germany).

Statistical analysis

For comparison of two samples calculation of statistical significance with two-tailed, two sample, equal variance student's t-test was done in Excel. For the gene expression analysis in the different DLBCL subtypes, two-way ANOVA was performed using GraphPad Prism.

Supplementary References

1. Sarchen V, Shanmugalingam S, Kehr S, Reindl LM, Greze V, Wiedemann S*, et al.* Pediatric multicellular tumor spheroid models illustrate a therapeutic potential by combining BH3 mimetics with Natural Killer (NK) cell-based immunotherapy. Cell Death Discov. 2022; 8: 11.

2. Schmitz R, Wright GW, Huang DW, Johnson CA, Phelan JD, Wang JQ*, et al.* Genetics and Pathogenesis of Diffuse Large B-Cell Lymphoma. N Engl J Med. 2018; 378: 1396-1407.

Supplementary Figure Legends

Supplementary Figure 1

DLBCL cell lines were exposed to A1331852 (RCK8: 3 nM, SUDHL8: 3 nM) with or without 50 μM of zVAD.fmk for 24 h before analysis of cell death using staining of phosphatidylserine (PS) with AnnexinV-FITC and flow cytometry.

Supplementary Figure 2

A) HBL1 cells were treated with ABT-199 or S63845 with and without zVAD.fmk (20 μM) for 20 h before protein lysis and analysis of caspase-3 and PARP cleavage. B-C) CRISPR/Cas9 mediated KO of caspase-9 was performed in SUDHL6 cells, and two single cell clones with deletion of caspase-9 were selected. B) Protein expression was assessed by Western blotting. C) Cell death induced by the BH3-mimetics ABT-199 (upper panel) or S63845 (lower panel) was investigated in gCtrl cells or caspase-9 KO clones at 24 h of treatment with different concentrations of BH3-mimetics. Data shown are mean +S.D. (n=3).

Supplementary Figure 3

A) SU-DHL-6 cells were treated with ABT-199 (3 μM for 24 h) or S63845 (300 nM for 16 h) with (grey bars) or without (black bars) zVAD.fmk (20 μM) before staining with MitospyGreen and flow cytometry. Data are presented as fold change compared to untreated control and mean + S.D. of four independent experiments. B) SU-DHL-6 or HBL-1 cells were treated with ABT-199 (3 μM) or S63845 (300 nM) for 24 h with or without zVAD.fmk (20 μM) followed by analysis of LC3 processing by Western blotting. GAPDH (SU-DHL-6) or Vinculin (HBL-1) were used as loading controls.

Supplementary Figure 4

Clones with Casp9 deletion (gCasp9 cl14 and cl16) and control cells (gCtrl) were treated with ABT-199 (3 μM) or S63845 (300 nM) for 16 h before differential lysis and analysis of

mitochondrial and cytosolic fraction by Western blotting (A) or staining with MitospyGreen and FACS analysis (B). Data are normalized to untreated control and shown as mean +S.D. (n=3).

Supplementary Figure 5

SUDHL6 cells were treated with ABT-199 (3 μM) or S63845 (300 nM) in the presence or absence of zVAD.fmk (20 μM) for 16 h before analysis of ROS using H2DCF staining or mitochondrial ROS using MitoSOX staining and flow cytometry. Data are normalized to untreated control and shown as mean +S.D. (n=3). B) Activation of intracellular signaling was analyzed by Western blotting. C) Ferroptosis was blocked using Ferrostatin-1 (Fer-1, 5 μM) or Liproxstatin-1 (Lip-1, 1 μM) with Erastin (5 μM) serving as positive control. Cell death was analyzed at 20 h of treatment using AnnexinV-FITC staining and flow cytometry. Data shown are mean +S.D. (n=3).

Supplementary Figure 6

SUDHL6 cells were treated with ABT-199 (3 μM) or S63845 (300 nM) in the presence or absence of zVAD.fmk (20 μM) for 16 h before analysis of mRNA expression using qRT-PCR. Data are normalized to untreated control and shown as mean +S.D (n=3).

Supplementary Figure 1

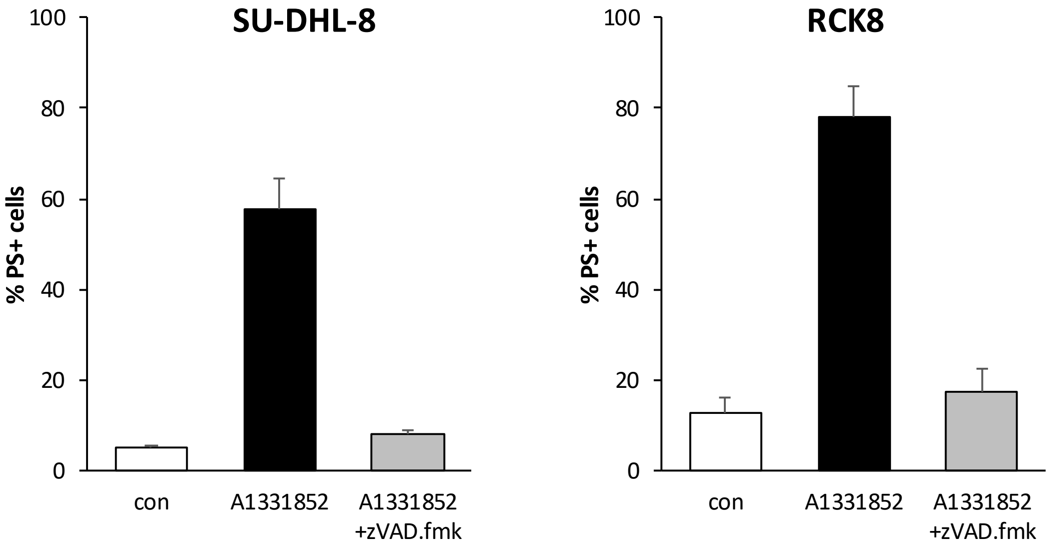

Supplementary Figure 2

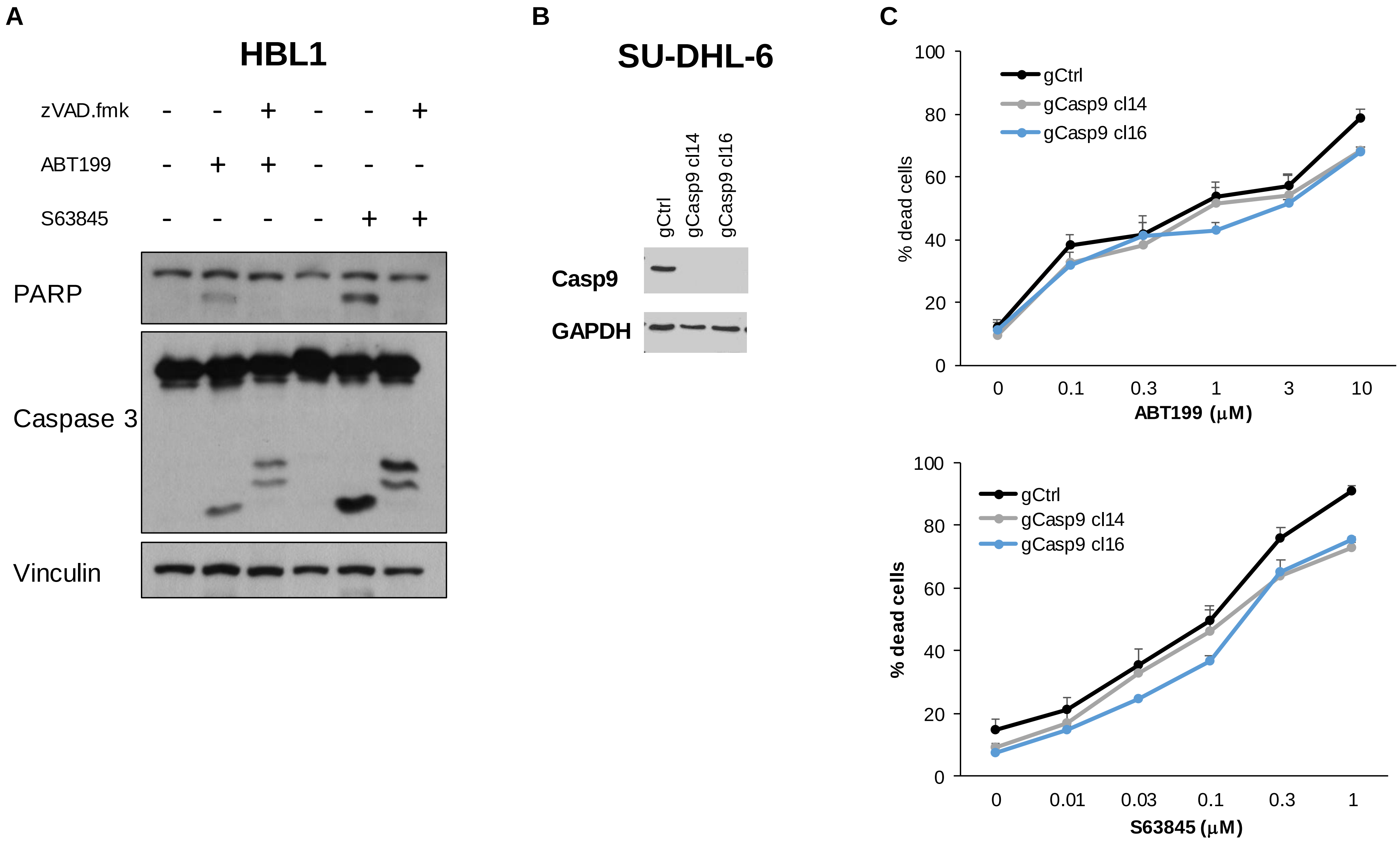

Supplementary Figure 3

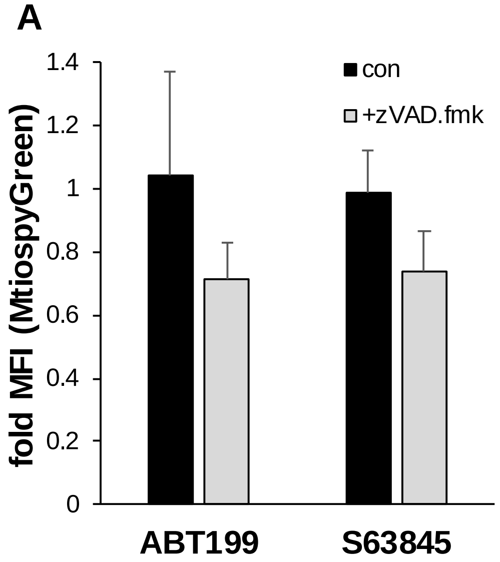

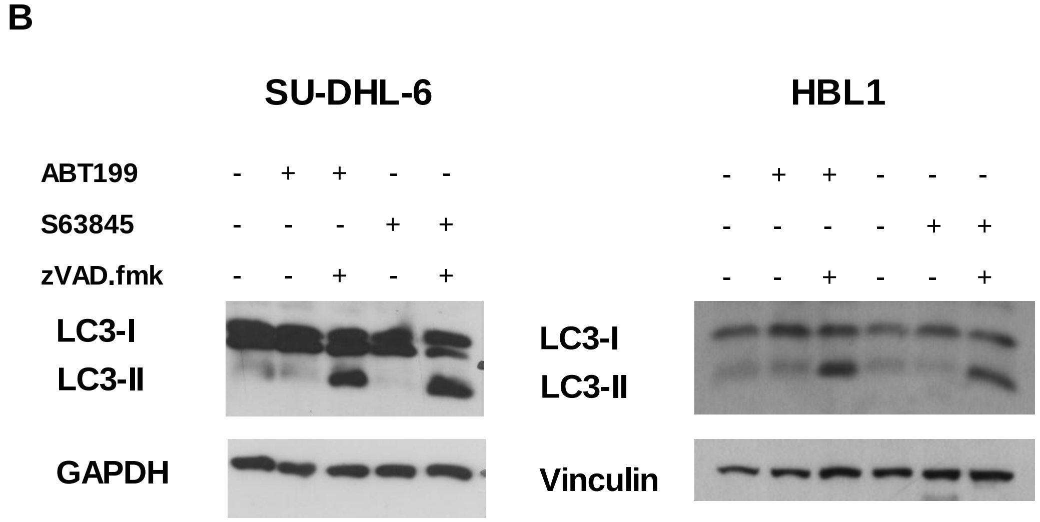

Supplementary Figure 4

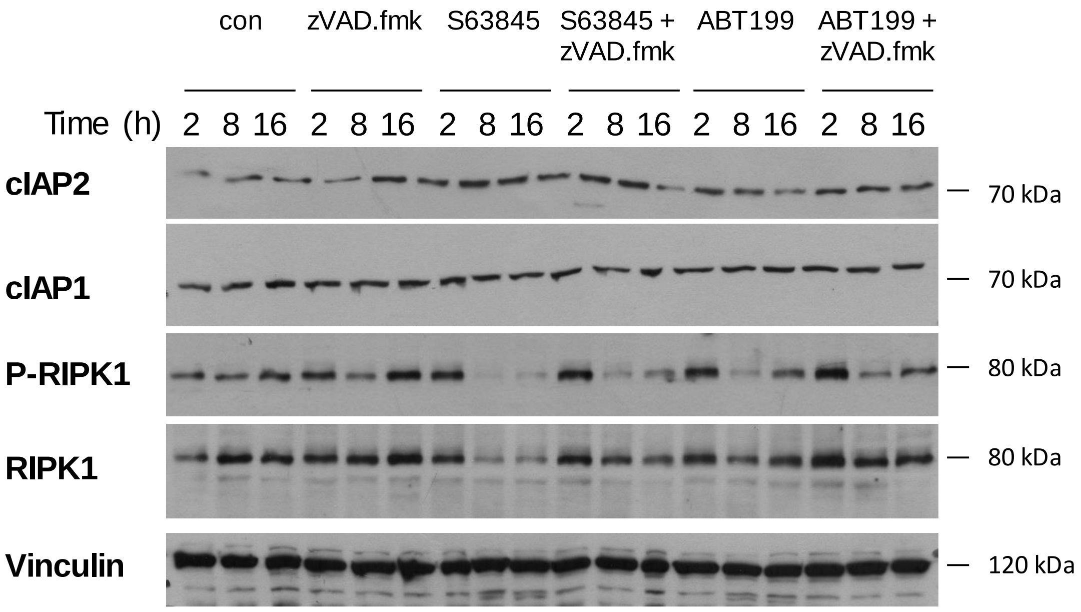

Supplementary Figure 5

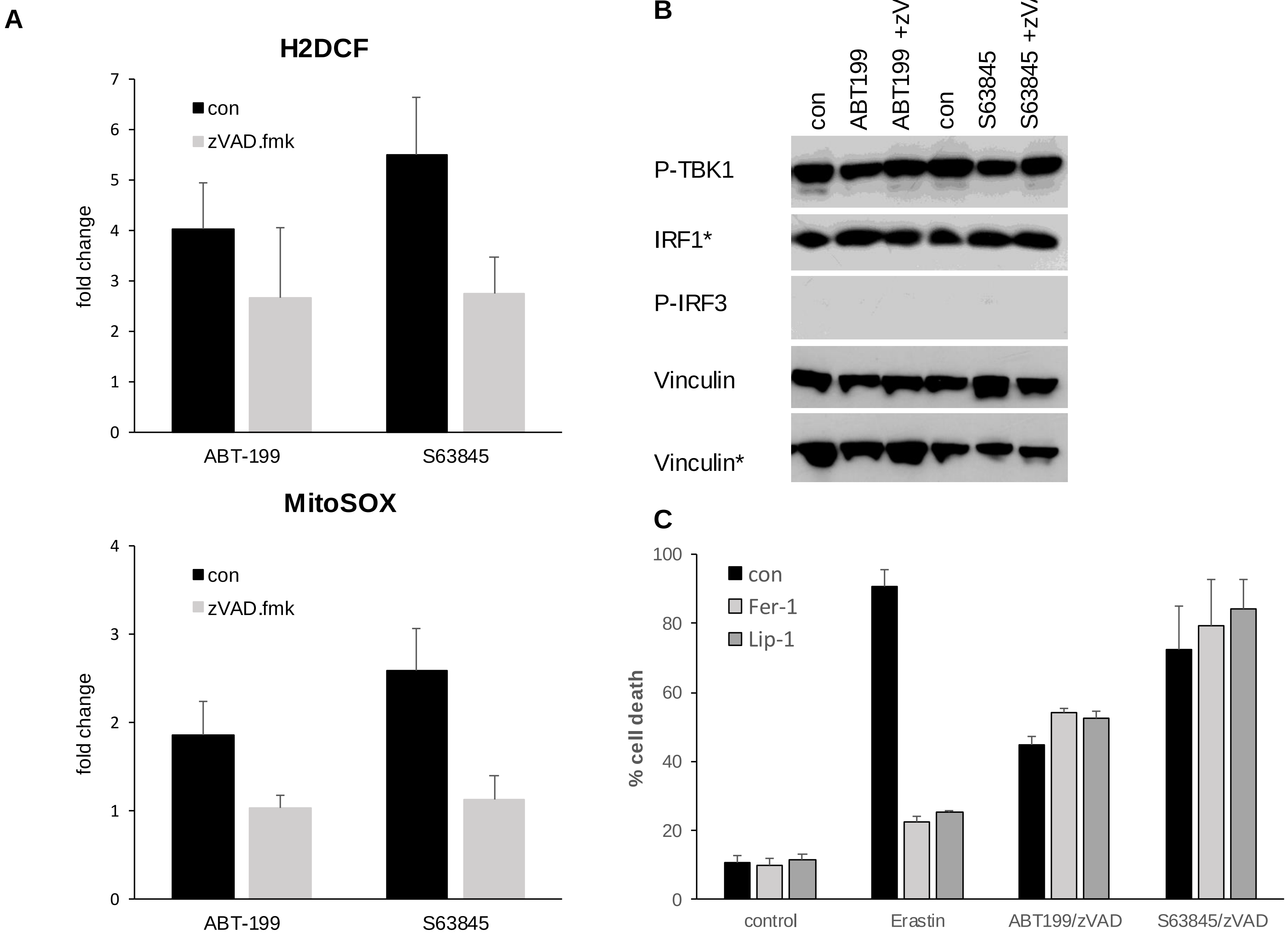

Supplementary Figure 6

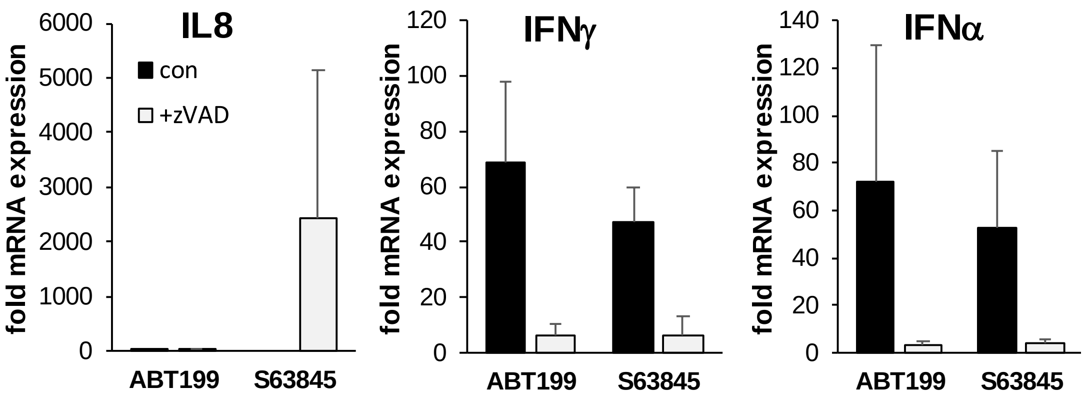

Supplement: Supplementary file 1 — Original Data File [file 41419_2024_6652_MOESM1_ESM.pdf]
