# Supplementary material for: Identification of a novel form of caspase-independent cell death triggered by BH3-mimetics in diffuse large B-cell lymphoma cell lines
Source: Cell Death Dis. 2024 Apr 15;15(4):266. doi: 10.1038/s41419-024-06652-3 (PMC11018778; doi:10.1038/s41419-024-06652-3)
Supplement: Supplementary file 3 — Original Data [file 41419_2024_6652_MOESM3_ESM.pptx]

## Slide 1
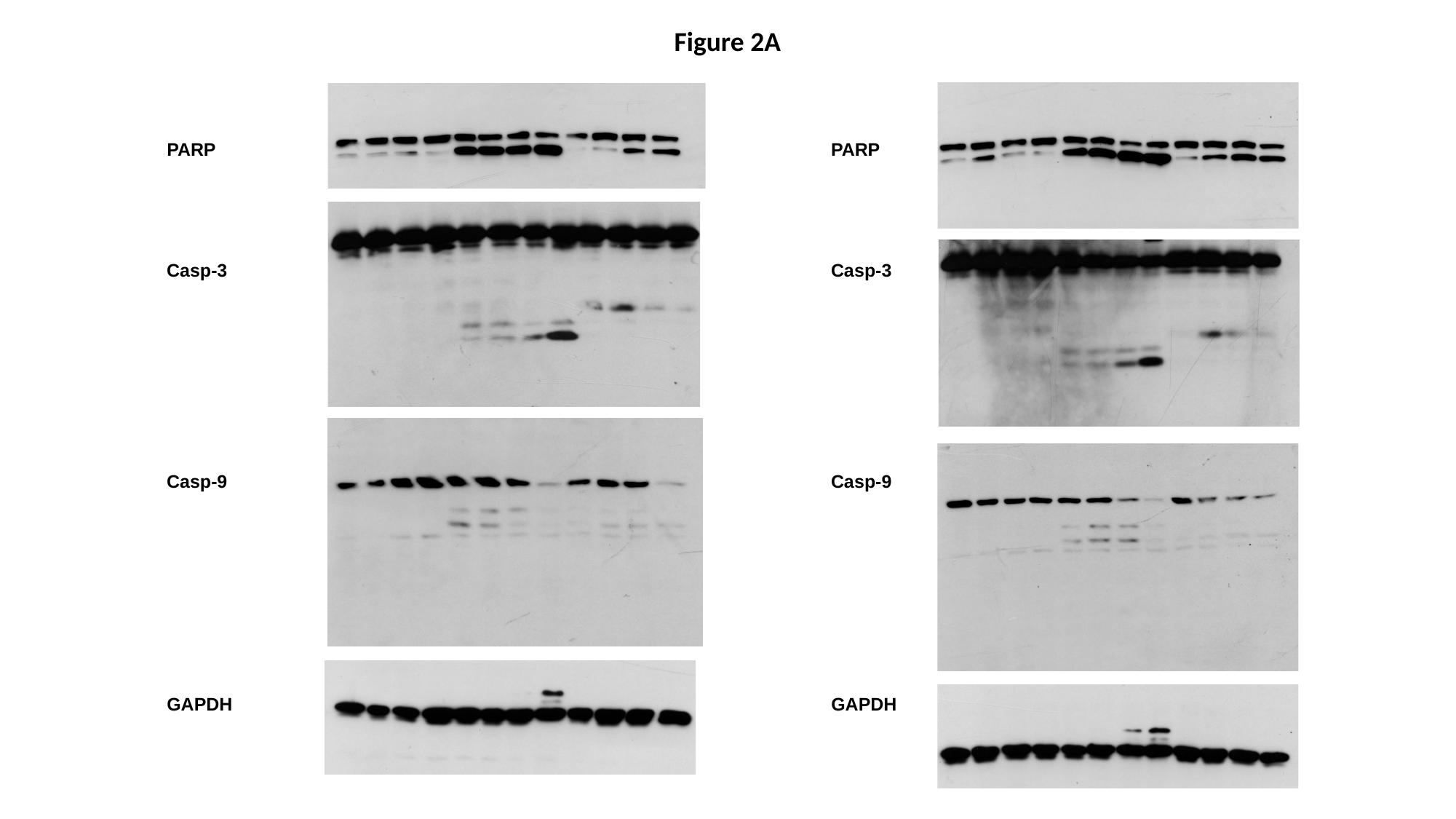

Figure 2A
PARP
PARP
Casp-3
Casp-3
Casp-9
Casp-9
GAPDH
GAPDH

## Slide 2
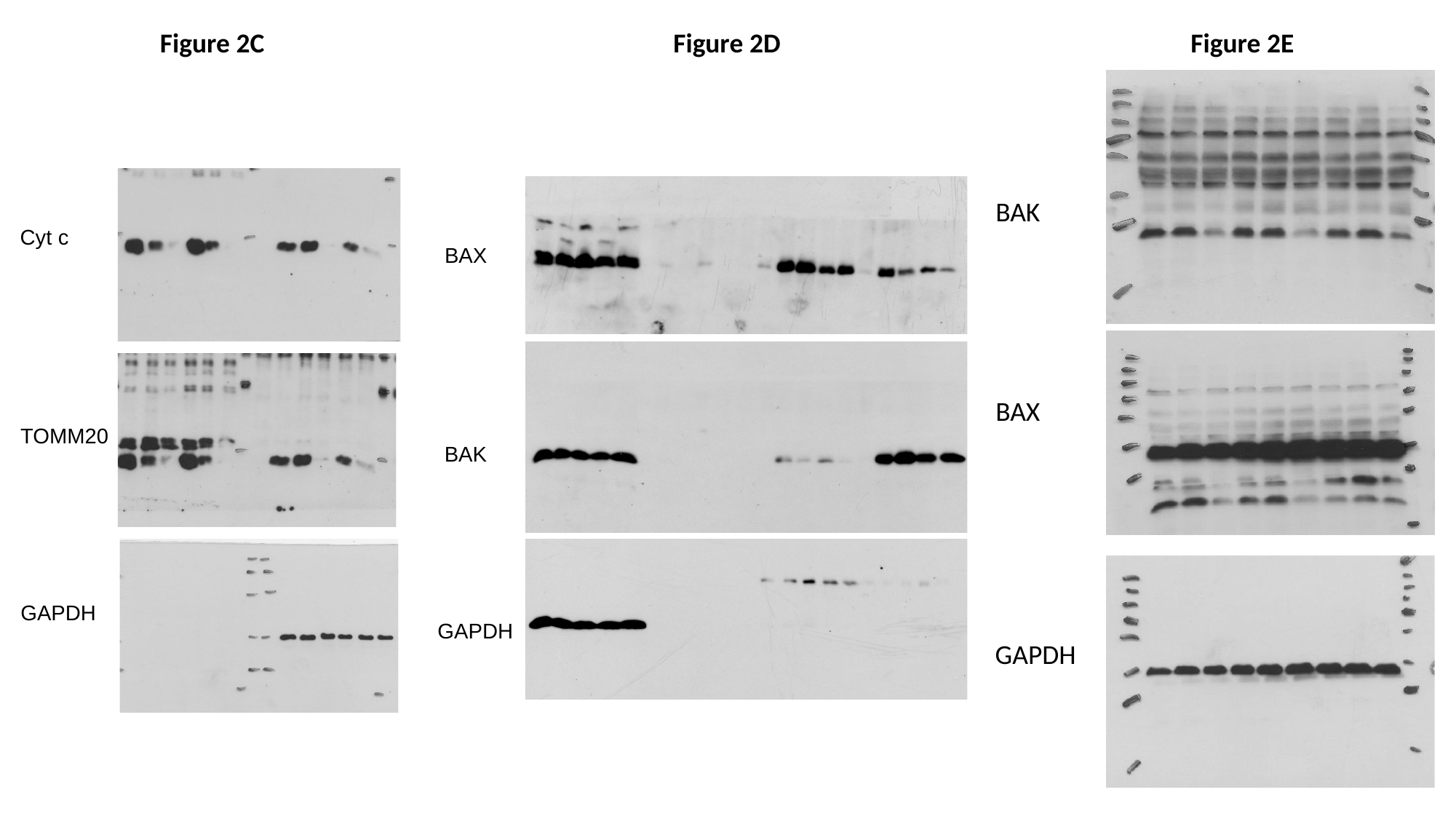

Figure 2C
Figure 2D
Figure 2E
BAK
Cyt c
BAX
BAX
TOMM20
BAK
GAPDH
GAPDH
GAPDH

## Slide 3
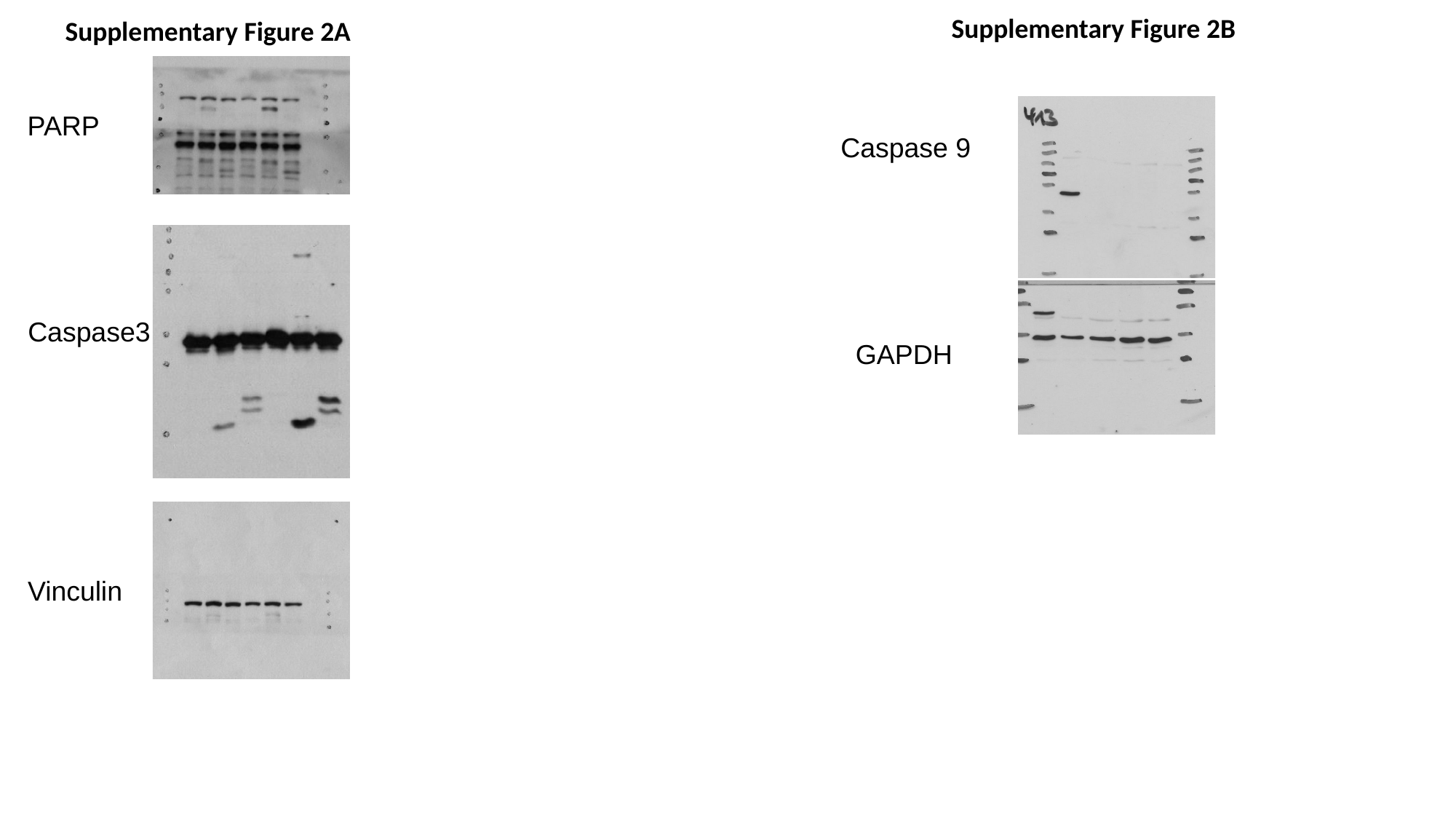

Supplementary Figure 2B
Supplementary Figure 2A
PARP
Caspase 9
Caspase3
GAPDH
Vinculin

## Slide 4
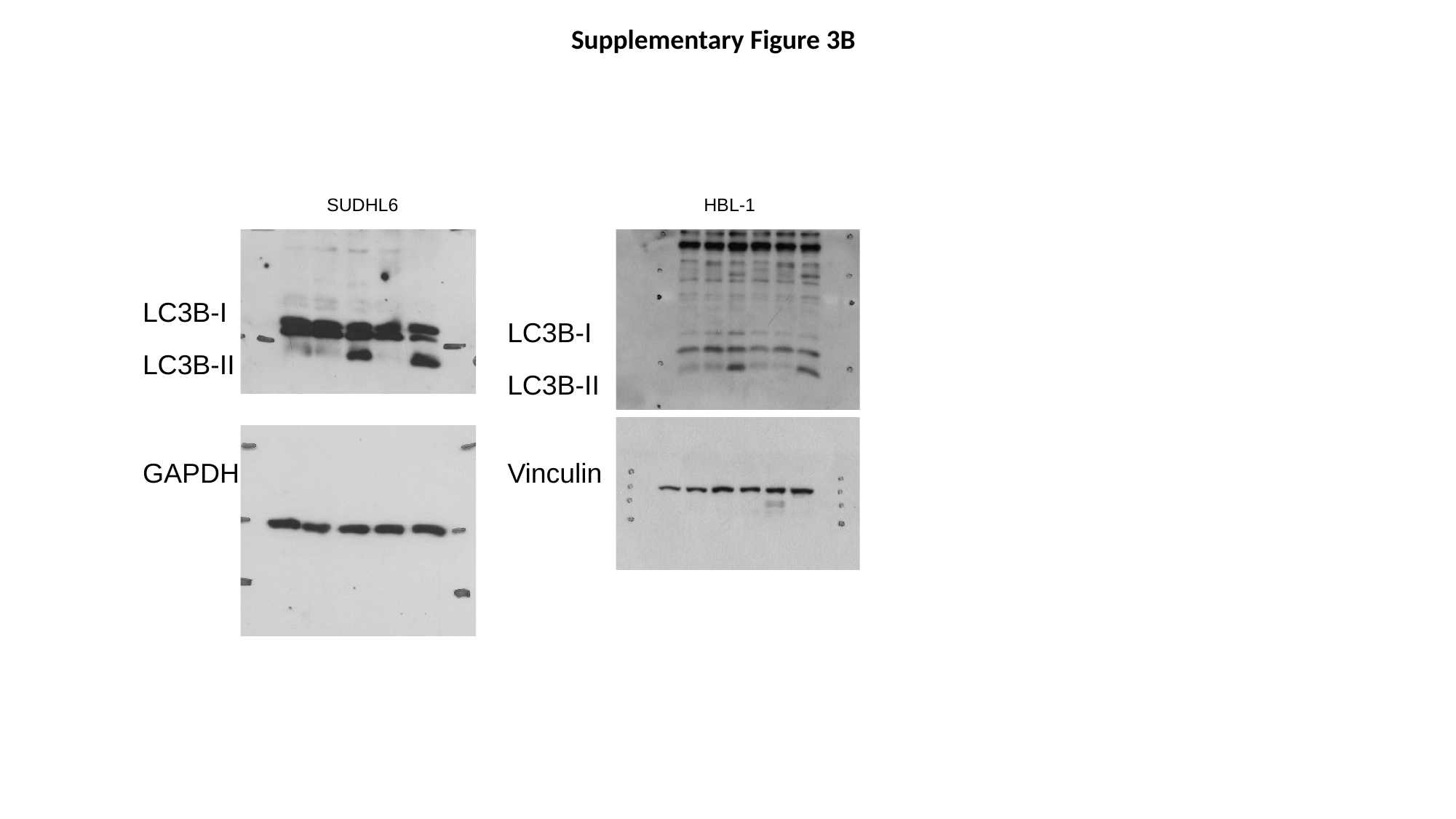

Supplementary Figure 3B
HBL-1
SUDHL6
LC3B-I
LC3B-I
LC3B-II
LC3B-II
Vinculin
GAPDH

## Slide 5
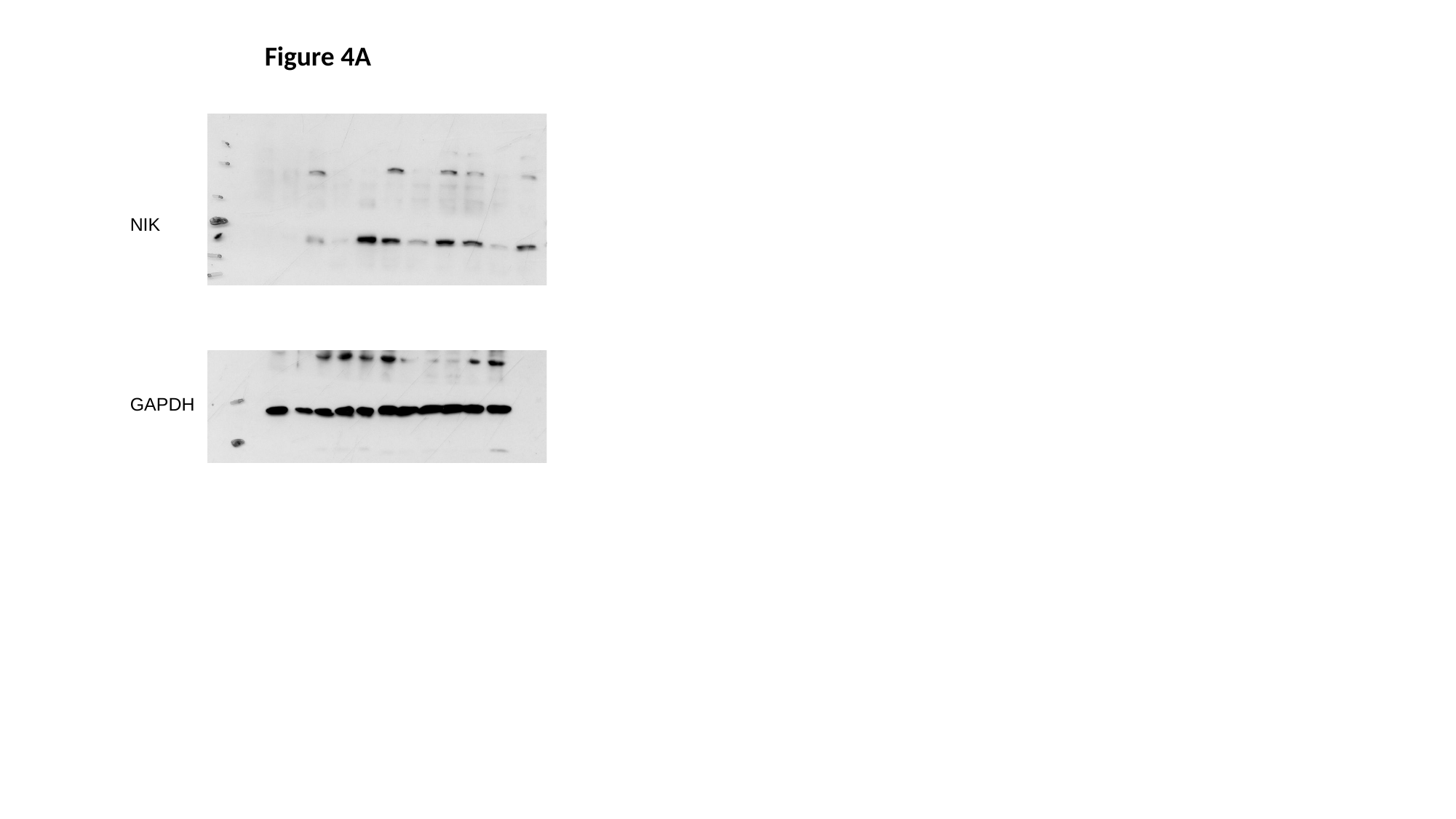

Figure 4A
NIK
GAPDH

## Slide 6
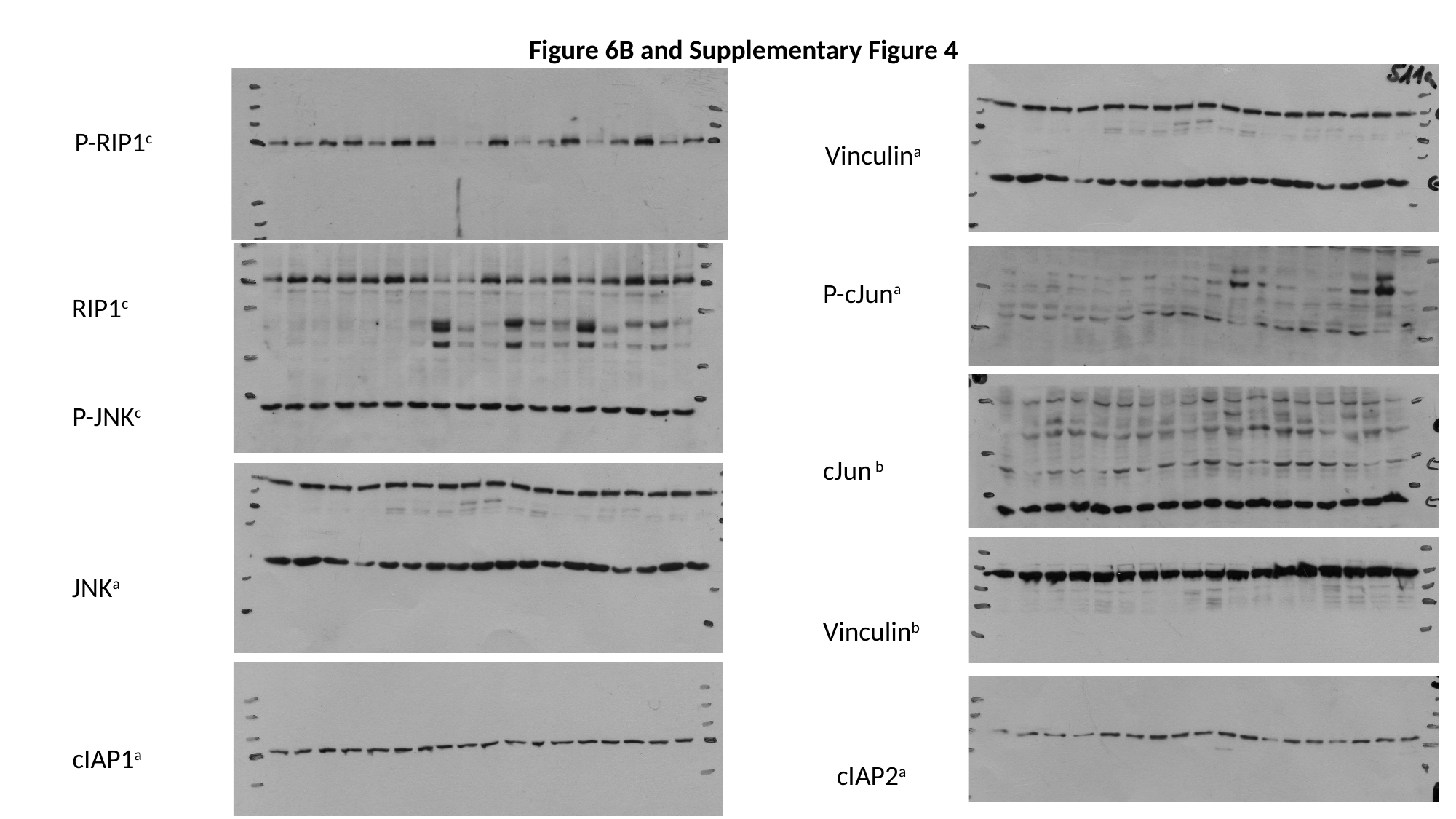

Figure 6B and Supplementary Figure 4
P-RIP1c
Vinculina
P-cJuna
RIP1c
P-JNKc
cJun b
JNKa
Vinculinb
cIAP1a
cIAP2a

## Slide 7
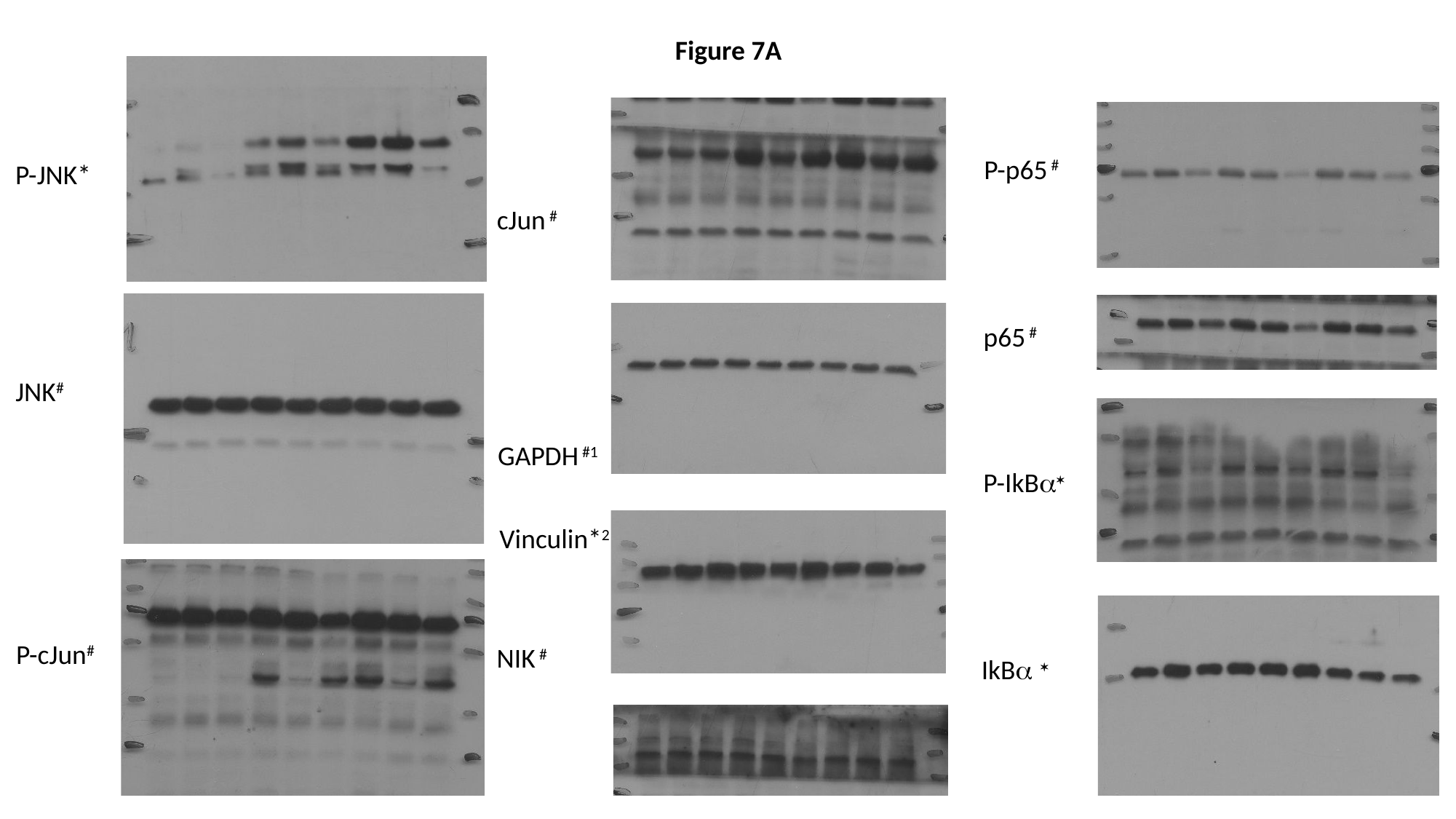

Figure 7A
P-p65 #
P-JNK*
cJun #
p65 #
JNK#
GAPDH #1
P-IkBa*
Vinculin*2
P-cJun#
NIK #
IkBa *
